# Gait variability and fatigability during a simulated 10-km running race in trained runners

**DOI:** 10.1007/s00421-025-05780-8

**Published:** 2025-04-21

**Authors:** Johnny Padulo, Marta Borrelli, Andrea Antiglio, Fabio Esposito

**Affiliations:** Department of Biomedical Sciences for Health (SCIBIS), Università Degli Studi di Milano, Via Colombo, 71, 20133 Milan, Italy

**Keywords:** Human locomotion, Footstep pattern coordination, Running performance, Endurance runners

## Abstract

**Purpose:**

Fatigue is an inevitable phenomenon during distance running, leading to the adoption of altered gait patterns by runners. Therefore, the aim of this study was to investigate the changes in gait variability and fatigability in distance runners over a 10-km running race.

**Methods:**

For this aim, 12 runners (36.5 ± 5.4 y) completed a simulated 10-km running race. Throughout the trial, heart rate (HR) and rate perceived exertion (RPE, CR-10) were analyzed. In addition, kinematic/kinetic measurements: contact time (CT), flight time (FT), step length (SL), stride time (ST) leg (k_vert_) and vertical (k_leg_) stiffness, were recorded. Gait variability including phase coordination index (PCI) was calculated for each 2-km segment.

**Results:**

HR increased (from 88.46 ± 5.84 to 93.87 ± 6.48 %HR_MAX_, *P* < 0.05) as well as RPE (6.58 ± 0.47–8.96 ± 0.40 a.u., *P* < 0.001) as the distance increased. Conversely, no differences between running segments for the kinematic/kinetic data (CT–FT–SL–ST–k_vert_–k_leg_) were observed over five 2 km. However, there was increase (*P* < 0.05) in gait variability (PCI), over the course of the running race. Furthermore, the determination coefficient (*R*^2^) was found as strong and very strong when compared five (0–10 km) and four (4–10 km) running segments between HR–RPE and PCI (*R*^2^ = 0.623–0.845).

**Conclusion:**

These results suggest that the gait variability is one mechanical determinant in assessing the neuromuscular output when the fatigability increases during a running race.

**Supplementary Information:**

The online version contains supplementary material available at 10.1007/s00421-025-05780-8.

## Introduction

Gait variability is a subject of growing interest in sport science, as it offers valuable insights into the underlying mechanisms of human locomotion, particularly in terms of bioenergetics and control. Numerous studies have investigated variability within the gait cycle paradigm for various forms of bipedal human gait, such as pedaling (Padulo et al. 2012), walking (Russo et al. 2020), race walking (Padulo et al. 2013), or running (Ardigò et al. 2023; Padulo et al. 2023b, a). Despite their distinct characteristics, these gait forms exhibit consistent and predictable movement patterns over time. The bipedal human gait is a multifaceted task that necessitates the lower limbs coordination and the orchestration of physiological muscle responses to accommodate natural or non-natural conditions (Plotnik et al. 2007). Individuals must adapt their footstep cycle to appropriately align with immediate environmental factors and achieve desired target values (Padulo et al. 2023 d).

Understanding the role of variability in the coordination and control of the sensorimotor system is of paramount importance in motor control research. Deviations in variability, either excessive or insufficient, during movement have been shown to adversely affect motor task performance (Davids et al. 2003). Expert athletes consistently exhibit reduced variability in kinetic and kinematics variables compared to less-skilled counterparts, indicating the significance of variability in achieving superior performance (Fleisig et al. 2009). Expert runners exhibit decreased variability in key variables such as step length and frequency, directly contributing to running speed, further supporting the importance of reduced outcome variability (Nakayama et al. 2010). Fatigue is a crucial factor influencing movement variability, as prolonged activity or muscle fatigue is associated with increased variability (Meardon et al. 2011). This phenomenon may be attributed to the role of movement variability in adapting to environmental perturbations, thereby preserving performance, as observed in studies on muscle fatigue during occupational tasks (Srinivasan and Mathiassen 2012). While the influence of fatigue on running has been extensively studied (Girard et al. 2013), the relationship between movement variability, movement outcome, and its modulation in response to increased fatigue during sports performance remains largely unexplored. Understanding the consistency of variability and its changes with escalating fatigue is crucial to determine when to measure an athlete’s typical variability during exercise. Variability values may alter within an exercise bout, especially when athletes experience fatigue or adapt to the task’s intensity as during 10-km running race (Renfree et al. 2020). Previous research has focused on analysing variability changes before and after fatiguing exercise (Gates and Dingwell 2011), but temporal changes during different phases of an exercise bout have not been thoroughly investigated. Therefore, the objective of this study was to analyze changes in variability during a 10-km running race. Based on previous research that indicates changes in variability with fatigue, it was hypothesized that variability would increase during a high-intensity continuous running protocol.

## Methods

### Participants

Twelve male runners (age 36.5 ± 5.4 years; body mass 71.7 ± 5.7 kg; body height 1.77 ± 0.06 m, BMI 22.96 ± 1.79 kg·m^−2^) voluntarily participated in this study. Inclusion criteria were training volume of more than 60 km per week, engaging in more than 3 training sessions per week, having performed at least three running races of 10 km in the last 6 months. The running training experience was 12 ± 8 years with 450 ± 129 min of weekly training. Furthermore, participants were required to be in good health without any neurological or musculoskeletal injuries. A statistical power of 80% (1–*β* = 0.80) and *α* = 0.05 were used to determine the necessary sample size of 12 participants. After being informed on the purpose and the procedures of the study, all the participants provided their written signed informed consent to participate in study that was approved by the local ethics committee and was performed in accordance with the principles of the latest version of the Declaration of Helsinki.

### Experimental design

Testing was carried over 2 days, with a 7-day interval. On the first day, anthropometric measurements were taken and familiarization with the 10-km circuit were performed. On the second day, after an individual tapering week (Mujika and Padilla 2003) to avoid any fatigue effects, each participant took part in a simulated running race of 10 km. During this trial, participants were asked, after a 10-min warm-up at self-selected speed, to run as if they were in a competitive 10-km race (usually the maximal speed was maintained throughout the distance). They were instructed to maintain regular water consumption within the trial, and water was provided ad libitum during the entire event. Verbal encouragement was given during the entire race. All procedures were performed on a 5-km asphalt circuit at 0.5% incline (average temperature 23.5 ± 3.5 °C, and relative humidity of 24.3 ± 1.7%) between 10:00 a.m. and 12:00 a.m., to ensure ecological validity. Each participant wore a GPS (Garmin Forerunner Rc730-XT, Garmin Ltd, Olathe, Kan), running clothing and personal shoes (Mizuno Wave Prodigy, Osaka, Japan) and a sport scientist rode alongside each runner on a bike equipped with a GPS (Garmin Forerunner Rc730-XT, Garmin Ltd, Olathe, Kan). Heart rate (HR, b·min^−1^) was continuously recorded during the trial (Polar H-10, Kempele, Finland), and it was normalized as a percentage of the maximal heart rate (208–0.7 × age), %HR_max_ (Tanaka et al. 2001). Participants reported their rating of perceived exertion (RPE, CR-10) immediately after each 2-km lap.

### Data analysis

At the same time, a foot-pod RunScribe^™^ system (Scribe Lab. Inc. San Francisco CA, USA) with a sampling rate of 500 Hz (precision of 0.002 s) attached to the lace shoe of the right and left legs, recorded the footsteps data (García-Pinillos et al. 2019). The foot-pod was calibrated before and re-checked after each trial. Results from RunScribe^™^ were taken from their website (https://dashboard.runscribe.com/runs) into the.csv file. Kinematic and kinetic parameters, including contact time (CT, ms), flight time (FT, ms), step length (SL, m), and stride time (ST, s) (calculated as the sum of CT (left and right) + FT (left and right)), as well as leg (k_leg_) and vertical (k_vert_) stiffness that were calculated according to Morin et al. (2005), were recorded. To analyze the gait variability of the stride duration, the phase coordination index (PCI) was calculated for every 2-km interval ensuring 600 footsteps (Meardon et al. 2011). In addition, the phase coordination index (PCI) was calculated from the FT-CT of both feet to assess running gait variability (Padulo et al. 2023c). Specifically, PCI was calculated by normalizing step time relative to stride time. Step time is defined as the time interval between the heel strike of one leg and the subsequent heel strike of the contralateral leg, while stride time refers to the time interval between the heel strike of one leg and the next heel strike of the same leg, as detailed in the Supplementary Material.

### Statistical analysis

Results are expressed as mean ± standard deviation (SD). Shapiro–Wilk test was used to verify the assumption of normality of the distributions. Repeated-measures analysis of variance (RM-ANOVA) was used to assess differences for HR, RPE, CT, FT, SF, SL, ST, k_vert_, and k_leg_, and PCI at every 2-km interval post-hoc analysis (LSD) between conditions was calculated. The effect size (ES) was also calculated (eta squared, $\eta_{p}^{2}$) for a better interpretation of the results (values of 0.01, 0.06 and above 0.15 were considered small, medium, and large, respectively (Cohen 2013). The coefficient of determination (*R*^2^) was calculated between HR–RPE (as proxy of the physiological effort) and gait variability (PCI), while all variables between over the distance intervals’ correlation. For the present study, we used the following interpretation criteria (Hopkins et al. 2009): very weak (*R*^2^ < 0.1), weak (*R*^2^ = 0.1–0.3), moderate (*R*^2^ = 0.3–0.5), strong (*R*^2^ = 0.5–0.7), very strong (*R*^2^ = 0.7–0.9) or extremely strong (*R*^2^ = 0.9–1). The significance level was fixed *P* ≤ 0.05 using Statistical Package for Social Science software (Version 28.0, IBM SPSS Statistics, Chicago, IL, USA).

## Results

The mean 10-km speed was 13.88 ± 1.55 km·h^−1^ (F_1,10_ = 4.377, and $\eta_{p}^{2}$ = 0.285 (ES: large) *P* = 0.028, Table 1) and HR 91.53 ± 6.34%HR_max_ with RPE of 7.87 ± 0.92 a.u. ANOVA showed no differences over five 2-km intervals running for the kinematic and kinetic data (post-hoc in Table 1), CT (F_1,10_ = 1.595, and $\eta_{p}^{2}$ = 0.127 (ES: medium) with *P* = 0.232), FT (F_1,10_ = 2.646, and $\eta_{p}^{2}$ = 0.194 (ES: large) with p = 0.122), SL (F_1,10_ = 2.353, and $\eta_{p}^{2}$ = 0.176 (ES: large) with *P* = 0.145), ST (F_1,10_ = 1.338, and $\eta_{p}^{2}$ = 0.108 (ES: medium) with *P* = 0.283), k_leg_ (F_1,10_ = 0.788, and $\eta_{p}^{2}$ = 0.073 (ES: medium) with *P* = 0.403), and k_vert_ (F_1,10_ = 1.486, and $\eta_{p}^{2}$ = 0.129 (ES: medium) with *P* = 0.253).

**Table 1:** Kinematic and kinetic variables and speed for each 2-km intervals

Variables	0–2 km	2–4 km	4–6 km	6–8 km	8–10 km	*R*^*2*^
CT (ms)	248 ± 17.53^†^	251 ± 16.13	252 ± 16.33	252 ± 15.71	250 ± 15.00	0.637
FT (ms)	104 ± 20.67^†^	102 ± 18.39^†^	100 ± 18.40	100 ± 17.85	100 ± 17.00	0.750
SL (m)	1.41 ± 0.12	1.39 ± 0.10^†‡^	1.38 ± 0.11	1.37 ± 0.11	1.37 ± 0.10	0.699
ST (sec)	0.707 ± 0.019	0.707 ± 0.018	0.706 ± 0.018	0.705 ± 0.019	0.713 ± 0.015	0.506
k_vert_ (kN·m^−1^)	19.58 ± 1.61	19.45 ± 1.46	19.44 ± 1.46	19.50 ± 1.41	19.77 ± 1.39	0.525
k_leg_ (kN·m^−1^)	8.00 ± 0.92	7.92 ± 0.88	7.95 ± 0.85^†^	8.03 ± 0.88^‡^	8.11 ± 0.90	0.506
Speed (km·h^−1^)	13.84 ± 1.54^†‡^	13.83 ± 1.60^†*^	13.92 ± 1.57	13.89 ± 1.57^‡^	13.98 ± 1.60	0.620

All data are expressed as mean ± standard deviation. Significant (*P* < 0.05) differences between the 1 st (0–2 km) and the 3rd (4–6 km) interval are denoted as “†”, while between 1 st (0–2 km) and 5 th (8–10 km) interval as “‡”. While significant (*P* < 0.05) differences between the 2nd (2–4 km) and the 3rd (4–6 km) interval are denoted as “†”, while between 2nd (2–4 km) and 4 th (6–8 km) interval as “‡”, and “*” between 2nd (2–4 km) and 5 th (8–10 km) interval. Significant (*P* < 0.05) differences between the 3rd (4–6 km) and the 5 th (8–10 km) interval are denoted as “†”, while between 4 th (6–8 km) and 5 th (8–10 km) interval as “‡”. The coefficient of determination (R2) between each variable on five distances’ intervals

**Fig. 1:**
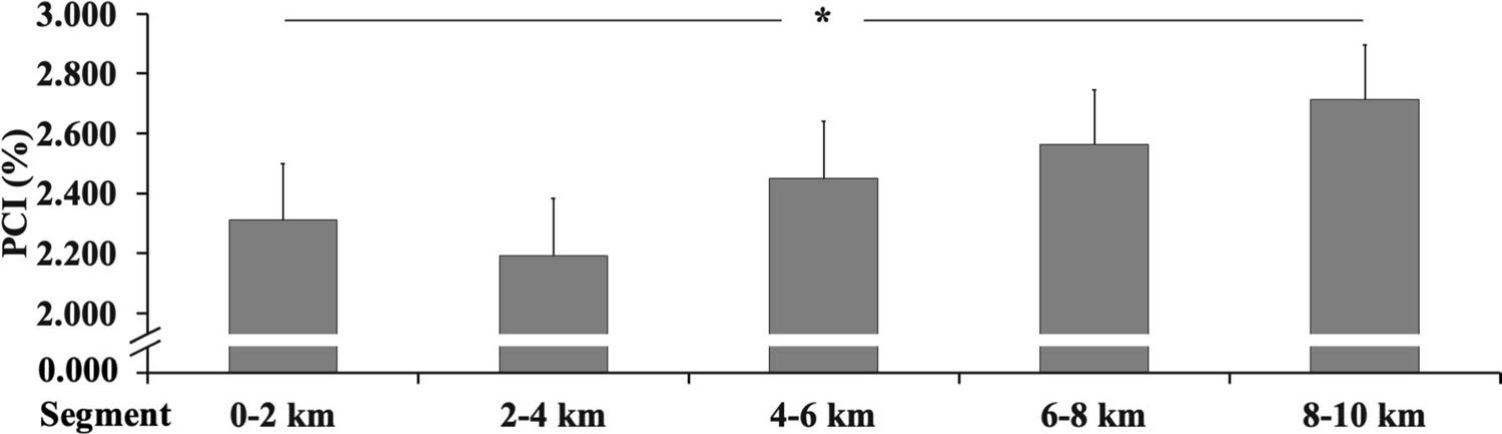
Running gait variability every 2 km during the 10-km running race. Phase coordination index (PCI). Values expressed as mean and standard error (“*”-*P* < 0.05)

Conversely, HR increased over the distance (F_1,10_ = 32.446, and $\eta_{p}^{2}$ = 0.747 (ES: large) with *P* < 0.001) as the RPE significantly increased (post-hoc in Table 2) when the distance increased (F_1,10_ = 103.013, and $\eta_{p}^{2}$ = 0.904 (ES: large) with *P* < 0.001). Further, gait variability (post-hoc in Fig. 1) was affected by the increased distance, PCI (F_1,10_ = 17.271, and $\eta_{p}^{2}$ = 0.611 (ES: large) with *P* < 0.001). The determination coefficient (*R*^2^) was found as strong and very strong when compared five (0–10 km) and four (4–10 km) distance levels between HR–RPE and PCI (*R*^2^ = 0.623–0.845).

**Table 2:** Heart rate and RPE for each 2-km intervals

Variables	0–2 km	2–4 km	4–6 km	6–8 km	8–10 km	*R*^*2*^
%HR_MAX_	88.46 ± 5.84^†^	90.67 ± 6.19	91.90 ± 6.40	92.77 ± 6.42	93.87 ± 6.48	0.821
RPE (a.u.)	6.58 ± 0.47^†^	7.58 ± 0.67	7.92 ± 0.42	8.29 ± 0.45	8.96 ± 0.40	0.893

All data are expressed as mean ± standard deviation. Significant (*P* < 0.01) differences in-between all interval conditions are denoted as “^†^”. The coefficient of determination (R^2^) between each variable on five distances’ intervals

## Discussion

The aim of this study was to compare the bilateral coordination, physiological and spatiotemporal parameters during the 10-km running race. The main finding indicated an increase in running gait variability and fatigability over the course of the race. Furthermore, the physiological response of running performance shows to be related to running gait variability. Our results, the optimal running speed, in terms of metabolic efficiency, seemed to be related to coordination factors, particularly in situations where impaired economy is difficult to justify.

Our study revealed that runners were able to maintain a consistent running speed with small variations (0.99 ± 0.01%). However, both HR and RPE increased throughout the 10-km race, reaching approximately 90 %HR_MAX_ and 90% for RPE. This aligns with findings by Damasceno et al. (2015) regarding metabolic demand, where the primary energy system contribution was shown to be 94% aerobic. Furthermore, well-experienced runners exhibited the ability to efficiently clear metabolites associated with fatigue during the 10-km race, while simultaneously engaging in both aerobic and anaerobic ATP resynthesis. Notably, as running speed increased, a biological stressor was introduced, resulting in a more predictable pattern of stride time variation (Jordan et al. 2007). This observation suggests that fatigue-induced alterations in running gait may contribute to the effects of increased stress on the body. Indeed, the impact of fatigue on bilateral coordination, as reflected in the accuracy and consistency of the phase relationship between the ST of the right and left legs (Fuller et al. 2017), cannot be overlooked. While locomotion speed has been widely used as a model to investigate the interference with central pattern generators, numerous findings have consistently demonstrated that the primary muscle activation patterns align with running speed (Cappellini et al. 2006). Thus, the running speed associated with the performance of well-experienced runners in the present study may provide insights into the utilization of optimized subcortical-level central pattern generators, elucidating the enhanced bilateral coordination (Hinman et al. 2018). The inter-strides bilateral variability is a component of bilateral coordination. Some studies have shown that gait variability is affected by fatigue (Fuller et al. 2017). However, to our knowledge, this study was the first to analyze the effect of constant running speed on bilateral coordination, specifically comparing physiological effort on 10-km running race.

**Fig. 2:**
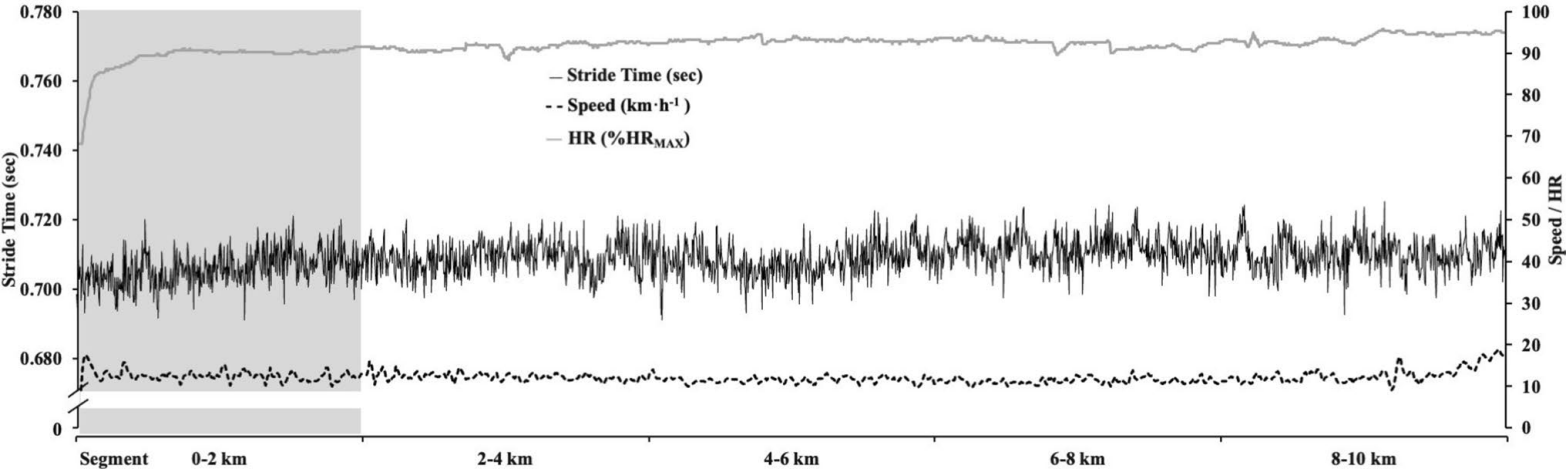
Representative plot of stride-to-stride duration during the 10-km running race; the figure show the speed and heart rate (HR)

Interestingly, the PCI can collectively inform a critical motor control factor related the constant speed as in 10-km running race where the metabolic demand is constant after the first kilometers (Table 2, Fig. 2). Moreover, also if the gait variability is higher (Fig. 1) in the first 2 km, we think that these results can be justified by several factors. The first factor is that each participant start to “0” speed to reach the maximal self-paced speed, usually of 2 km (Renfree et al. 2020) with large speed variations. At the same time, the kinematic and kinetic data showed a different trend respect to 2–4 km intervals, to support this critical factor. Moreover, in the first 2 km, there is a second factor influencing the footstep analysis related to the high increased ~ 30% metabolic expenditure (Fig. 2) from start to “0” speed to reach the maximal self-paced speed. All these factors can be related to different tasks (Hamill et al. 1999), thus to reach the optimal speed that can be maintained for all the distances. Furthermore, the standard procedure of gait variability requires constant environmental conditions; therefore, the gait variability cannot be considered in the first 2 km. Indeed, when the individual optimal speed is reached, the participants maintained throughout the distance. In this condition as in our study, the strategy (Abbiss and Laursen 2008) by the participants was to maintain the maximal speed (~ 90 %HR_MAX_) reached at 2 km till 10 km. These findings are supported by the coefficient of determination (*R*^2^) when compared 0–10-km and four 4–10-km distance levels between HR–RPE (as physiological effort) and gait variability as strong/very strong for PCI (*R*^2^ = 0.623–0.845). Therefore, the current study highlights the influence of fatigue (as physiological effort) on gait variability during the running race.

Gait variability may contribute to understanding the mechanisms involved in self-selected running pace choice and metabolic optimization in well-experienced runners. The phenomenon observed in this study can be attributed to various underlying mechanisms. Muscle orchestration and synchronization among different systems provide a plausible explanation for our findings. In particular, changes in muscle activity weight under fatigue conditions appear to be a compensatory strategy for maintaining muscular force output (Hajiloo et al. 2020). These changes may reflect the central nervous system’s approach to optimizing motor system function. In addition, increased coherence between the cardiovascular and muscular systems has been linked to a self-selected high-speed running stride rate, resulting in enhanced metabolic performance (Carvalho et al. 2021). These mechanisms contribute to the observed performance benefits and provide insight into the interplay between physiological systems during running. In line with prior reports on fatigue, the notable linear tread indicates a general decline in long-range correlations during the run (Gates and Dingwell 2008). One plausible interpretation offered for this decrease is the heightened necessity for adjustments as fatigue sets in, possibly due to the loss of motor unit firing and force production, along with increased variability in motor unit firing during fatigue (Selen et al. 2007).

## Conclusions

These results suggest that gait variability serves as a mechanical indicator for assessing the neuromuscular output as fatigability increases during a running race. As gait variability increased in relation to physiological effort, it may be utilized as a useful metric to evaluate the impact of fatigue on neuromuscular function during such endurance events.

### Practical implications

- This study shows bilateral coordination as an extremely useful assessment method in the daily life of running coaches and runners (i.e., reached lower PCI when the musculoskeletal load capacity will be able to after a chronic load adaptation as in steady-state condition).
- We showed the potential use of this innovative parameter (PCI) of running motor control.
- The bilateral coordination used in this study can be assessed with a minimum number of analyzed strides, thus being feasible for day-to-day training and during different race phases.

## Supplementary Information

Below is the link to the electronic supplementary material.

Supplementary file 1 (DOCX 36 KB)

## Data Availability

The data are available upon specific and reasonable request through direct contact with the corresponding author.

## Abbreviations

ANOVA: Analysis of variance
CNS: Central nervous system
CT: Contact time
FT: Flight time
HR: Heart rate
k_leg_: Leg stiffness
k_vert_: Vertical stiffness
PCI: Phase coordination index
RPE: Rating of perceived exertion
SF: Step frequency
SL: Step length
ST: Stride time
$${\dot{\text{V}}}$$ O_2_: Oxygen consumption
%HR_MAX_: Percentage of the maximal heart rate
