# Correction: Gait variability and fatigability during a simulated 10-km running race in trained runners

**DOI:** 10.1007/s00421-025-05873-4

**Published:** 2025-07-30

**Authors:** Johnny Padulo, Marta Borrelli, Andrea Antiglio, Fabio Esposito

**Affiliations:** Department of Biomedical Sciences for Health (SCIBIS), Università Degli Studi di Milano, Via Colombo, 71, 20133 Milan, Italy

**Correction: European Journal of Applied Physiology** 10.1007/s00421-025-05780-8.

In the original version of this article, under the Abbreviations section, SF Step Frequency should be deleted and in ‘’statistical analysis’’ section SF should be deleted.

