# Supplementary material for: Gait variability and fatigability during a simulated 10-km running race in trained runners
Source: Eur J Appl Physiol. 2025 Apr 21;125(9):2529–35. doi: 10.1007/s00421-025-05780-8 (PMC12423248; doi:10.1007/s00421-025-05780-8)
Supplement: Supplementary file 1 — (DOCX 36 KB) [file 421_2025_5780_MOESM1_ESM.docx]

**Supplementary Material**

To analyse the bilateral coordination the phase coordination index (PCI) was calculated according to the Plotnik’s equation (2007).

We assessed left-right coordination in walking gait using the PCI, following the method described by Plotnik and colleagues (2007). This index involved normalizing step time in relation to stride time. Step time refers to the time interval between a heel strike and the subsequent one of the contralateral leg, while stride time is the time interval between a heel strike and the consecutive one of the same leg. The normalization of step time with respect to stride time allowed us to calculate the phase (*ϕi*) of each stride, serving as an index of bilateral coordination (Plotnik et al. 2007). To ensure consistency of participant, regardless of potential dominance differences, we calculated the average step time for both legs and used the leg with the longer step time as the reference for gait cycles. Subsequently, we computed *ϕi* values for the other leg using the formula:

*ϕi* = 360° × $\frac{\boldsymbol{t}_{\boldsymbol{Si}}\boldsymbol{-}\boldsymbol{t}_{\boldsymbol{Li}}}{\boldsymbol{t}_{\boldsymbol{L}\boldsymbol{(}\boldsymbol{i}\boldsymbol{+1)}}\boldsymbol{-}\boldsymbol{t}_{\boldsymbol{Li}}}$ (1)

where *t_Si_* and *t_Li_* denote the time of the *i*-th heel strike of the legs with the short and long ST, respectively, and *t_L(i+1)_* > *t_Si_* > *t_Si_*.

The denominators in equation (1) correspond to the step time (ST) of the leg with the longest step time. Furthermore, we applied a conversion factor of 360 to transform the variable into degrees. A *ϕ* value of 180° signifies successful walking symmetry, where step time constitutes half of the gait cycle for each step. The evaluation of the accuracy and consistency of phase generation is encompassed by running gait variability, serving as the primary outcome. To assess the accuracy level in phase generation, measuring how closely the series of generated phases align with the value 180°, we calculated the mean value of the absolute differences between the phase at each stride and 180°. This measure is denoted as *ϕ*_ABS:

*ϕ*_ABS [°]= $\bar{\left| \boldsymbol{\phi}_{\boldsymbol{i}}\boldsymbol{-180^{\circ}} \right|}$.

To assess the level of consistency in phase generation across all strides for each participant, we calculated the coefficient of variation of the mean of *ϕ*, representing this consistency as *ϕ*_CV [%]. To account for the relationship between *ϕ*_ABS and *ϕ*_CV, we derived the phase coordination index (PCI) as follows: PCI = *ϕ*_CV + P*ϕ*_ABS, where P*ϕ*_ABS = 100 × (*ϕ*_ABS/180). Additional details regarding the association between *ϕ*_ABS and *ϕ*_CV can be found in the work by Plotnik and colleagues (2007). Notably, PCI provides insights into both the accuracy and consistency of phase generation.

**References**

Plotnik M, Giladi N, Hausdorff JM (2007) A new measure for quantifying the bilateral coordination of human gait: Effects of aging and Parkinson’s disease. Exp Brain Res 181:561–570. https://doi.org/10.1007/s00221-007-0955-7
